# Legacies to Medical Men by Their Patients

**Published:** 1910-02-05

**Authors:** 


// The Hospital

A Journal of

The Medical Sciences and Hospital Administration.

New Series. No. 155, Vol. VI. [No. 1227, Vol. XLVII.] SATURDAY, FEBRUARY 5, 1910.

### Legacies to Medical Men by their Patients.

The case of Rawnsley *v.* The *British Medical Journal*, which came before the Lord Chief Justice and a Special Jury on January 24, although one of libel, really arose out of an action brought by the plaintiff to upset the will of his wife, made in favour of her medical attendant, Dr. William Dunn. The plaintiff in February of last year brought an action with respect to his wife's will, alleging that Dr. Dunn had obtained it by undue influence. A settlement was arrived at, by which Dr. Dunn gave up the greater portion of his benefits under the will. The *British Medical Journal*, in commenting on the case, said: "Dr. William Dunn, now medical officer to Uppingham School, but formerly in practice in Battersea, had among his patients there a lady who, when she first came under his care, was living apart from her family in very poor circumstances. Besides advising her as to her health, Dr. Dunn lent her money, and Mrs. Dunn tried to make peace between her and those from whom she was estranged. Later the lady inherited a considerable sum of money from a sister, and then she seems to have begun making a number of different wills. . . . There was no proof that any undue influence was exercised either by Dr. Dunn or his wife. . . . We especially congratulate Dr. Dunn on the virtual acquittal from an odious charge which the settlement involves." The plaintiff went to the defendants and pointed out that they were wrong in their facts. The plaintiff was not satisfied with the apology the defendants inserted in their paper, and therefore brought the present action, as he considered the articles implied that he had left his wife poor and friendless. The defendants called no evidence, and said an honest slip had been made, and there was no malice. The jury awarded the plaintiff £750 damages.

A strong case must be made out to set aside a will on the ground of undue influence; nor is it sufficient to prove mere influence, but there must be such a degree of influence as deprives the testator of being the proper master of his faculties. Where a will is executed by an old man of weakened capacity in favour of his medical attendant there must be the clearest proof, not only of the factum of the instrument, but of the testator's knowledge of its contents. In Durnell *v.* Corfield (3 L. T (o.s.) 323) the deceased left behind him three testamentary papers—namely a will executed in November 1840, another will executed in September 1841, and a codicil dated November 21, 1843, nine days only prior to his death. The will of 1841 revoked that of 1840, and the codicil of 1843 purported to revive the will of 1840. The effect of the codicil was to give the bulk of his property, about £1,200, to the medical attendant of the deceased (on condition that he should take care of his only sister during her life), and to appoint him executor and trustee, though the wording of the document implied an intention that it should operate during the life of the deceased. The deceased at the time of his death had reached the age of 80 years. The medical man was excluded from probate, the Court not being satisfied that the deceased knew of the contents of the will and approved of it. Where a physician drew up a will wherein he was appointed executor and residuary legatee for a female patient of undoubted capacity, but suffering under a complication of disorders, which terminated her life within six days, the Court held that the words constituting the residuary clause were not entitled to probate, the evidence not being sufficiently clear that the bequest was contained in the will at the time of its execution, and that the testatrix knew that it was so. It was proved in this case (Greville *v.* Tyler, 7 Moo. P.C. 320) that the testatrix had often spoken of her relations as if she hated them, and declared that they should not have her money, but that her physician should have, at her death, all she possessed. Lushington, in delivering the judgment of the Privy Council, said: "There can be but little doubt that the appellant (the physician) has failed to derive from the testatrix's will benefits which, of some kind, and of some amount, she really intended him to take. But he has nobody but himself to blame. If a physician after a long attendance on a patient, thinks fit, when she is almost on her death-bed, to prepare and procure the execution of a will, by which he becomes the principal object of her bounty, to the exclusion of her near relations, and to do this without the intervention of any solicitor, or other person competent to give her advice, and to guard her against undue influence, the interests of the public require that his conduct should be regarded by courts of justice with the utmost jealousy."

There is nothing in the relation of medical attendant and patient which can prevent the one from entering into a contract with the other, but while such relation continues, the Court will look upon a contract entered into without the intervention of a third party with considerable jealousy.

